# Buffalo City Hospital

**Published:** 1846-11

**Authors:** 


*Buffalo City Hospital.*—At Meetings of several citizens on the 23d and 26th ult., held at the office of Henry W. Rogers, Esq., Collector of the Port of Buffalo, an Association was formed for the establishment of a Public Hospital in this city. The association consists, at present, of 35 directors, and the intention is to apply to the next Legislature for an act of incorporation, and pecuniary endowment. The claims of this city, from its peculiar position as a commercial town, to an appropriation from the State for the relief of non-residents and emigrants, who become subjects of public charity in sickness, are of a character which can hardly be resisted, when similar claims of New-York city have always been so fully recognised. The City Hospital of New-York has received from the State since its organization nearly a million of dollars, and is now maintained chiefly by a state annuity of twelve thousand five hundred dollars. We trust that our claims will be urged, and responded to during the next Legislative Session.

The officers elected by the association are are follows :—

*President*, JOSIAH TROWBRIDGE, M. D.

1*st Vice President*, Gen. H. B. Potter.

2*d Vice President*, Geo. W. Clinton, Esq.

*Secretary*, E. S. Baldwin.

*Treasurer*, S. N. Callender.

*Executive Committee*,—R. H. Heywood, Bryant Burwell, M. D., and George Jones.

*Committee to make application to the Legislature*,—Henry W. Rogers, Geo. W. Clinton, and F. H. Hamilton.

Officers of the Hospital for the ensuing year :—

*Attending Surgeon*,—F. H. Hamilton, M. D.

*Attending Physician*,—Austin Flint, M. D.

*Counselling Phys. and Surgs.*—Drs. Trowbridge and Burwell.

It is contemplated that this Institution will embrace a Marine Hospital department. The Attending Physicians and Surgeons to this department are, S. G. Bailey, M. D. and Austin Flint, M. D.

